# Multilabel segmentation of cancer cell culture on vascular structures with deep neural networks

**DOI:** 10.1117/1.JMI.7.2.024001

**Published:** 2020-04-07

**Authors:** Samuli Rahkonen, Emilia Koskinen, Ilkka Pölönen, Tuula Heinonen, Timo Ylikomi, Sami Äyrämö, Matti A. Eskelinen

**Affiliations:** a University of Jyväskylä, Faculty of Information Technology, Jyväskylä, Finland; b Tampere University, Faculty of Medicine and Health Technology, Finnish Centre for Alternative Methods, Tampere, Finland

**Keywords:** neural network, segmentation, cancer, *in vitro*, microscopy

## Abstract

New increasingly complex *in vitro* cancer cell models are being developed. These new models seem to represent the cell behavior *in vivo* more accurately and have better physiological relevance than prior models. An efficient testing method for selecting the most optimal drug treatment does not exist to date. One proposed solution to the problem involves isolation of cancer cells from the patients’ cancer tissue, after which they are exposed to potential drugs alone or in combinations to find the most optimal medication. To achieve this goal, methods that can efficiently quantify and analyze changes in tested cell are needed. Our study aimed to detect and segment cells and structures from cancer cell cultures grown on vascular structures in phase-contrast microscope images using U-Net neural networks to enable future drug efficacy assessments. We cultivated prostate carcinoma cell lines PC3 and LNCaP on the top of a matrix containing vascular structures. The cells were imaged with a Cell-IQ phase-contrast microscope. Automatic analysis of microscope images could assess the efficacy of tested drugs. The dataset included 36 RGB images and ground-truth segmentations with mutually not exclusive classes. The used method could distinguish vascular structures, cells, spheroids, and cell matter around spheroids in the test images. Some invasive spikes were also detected, but the method could not distinguish the invasive cells in the test images.

## 1 Introduction

In many cases, chemotherapeutic anticancer therapies’ contribution to life extension is low, and serious adverse effects are common.^1^^,^^2^ Also the cost of cancer treatments is extremely high and is increasing constantly.^2^

To improve the clinical benefits of treatments, there is a need to find new ways to treat cancer and to investigate which drugs or drug combinations are the most effective for individual cancers and patients. This could be achieved by developing new *in vitro* models that are able to reliably predict the effect of drugs *in vivo*. To get more relevant and reliable information from *in vitro* techniques, *in vitro* cancer research has been increasingly interested in alternative models that better mimic the tumor environment *in vivo*.^3^ These new models are often complex and have many parameters that need to be considered simultaneously. Therefore, there is a need for computational methods to recognize and quantify these new parameters and handle the massive amount of data with high throughput.

In this study, we cultivated prostate carcinoma cell lines PC3 and LNCaP on top of a matrix with vascular structures. We were able to identify some characteristics, such as structures and appendages, which are thought to represent invasiveness and possible metastatic potential. A phase microscope was used for taking the images. The PC3 cells lined up with the vascular structures and could be seen as lined-up matrix-covering networks at later time points. The LNCaP cells grew in spheroids that in the last imaging days showed spike-like formation growing from their edges with cells and cell matter surrounding the spheroids. Changes in spheroids were demonstrated to correlate with gene expression patterns in studies by other research groups.^4^^,^^5^

Segmentation is a technique to extract additional features from images, which can be used for further analysis. Image segmentation means classifying images at the pixel level. In this case, the microscopy images of cell cultures could be classified per pixel to different cells and structures.

The limitation of the used imaging method is that it is difficult to distinguish different cells from each other. Staining the cells would make identification easier, but the available staining options could damage or even kill the cells in addition to increasing expenses and labor. Disturbances to cells change their movements and surface proteins enough to influence the results. Also, the fluorescence stains might have unknown and unwanted interactions with cancer drugs. This prevents the use of the common solution in literature in which fluorescence markers would be used as segmentation labels.^6^ Fluorescence technology requires excitation, which also needs to be avoided due to possible interference with later applied study chemicals and drugs.^7^

Further feature engineering could reveal new information on how different cells and structures interact. Possible features are, for example, the morphological features, such as the area, perimeter, and diameters of different cells.^6^ Also using distances between different cells and structures could be used to identify spatial and functional relationships.^8^ For example, Ref. 9 Ahonen et al. measured how different drug treatments can have an effect on the area and roundness of cells. Assessment of the drug efficacy could be achieved similarly after the structures were segmented first.

Six classes of structures, which were thought to include meaningful data for testing the drug efficacy, were identified in the images. Since the structures are semitransparent and may overlap, we are considering a multilabel problem with mutually not exclusive classes. Therefore, the proposed solution approaches the problem by carefully constructing datasets for training multiple specialized U-Net neural networks^10^ to detect the classes from microscopy images.

## 2 Related Work

Segmentation is used widely in medical image analysis. Many past applications are based on traditional image processing algorithms to produce segmentations tailored to the problem. For example, Ref. 9 used the local entropy filter and Watershed algorithms for thresholding cell culture images and a support vector machine for segmentation. The structures were extracted from the image background and separated from each other. The texture features were engineered based on, e.g., the local binary pattern histogram and Haralick features.

Deep neural networks (DNNs) do not need such feature engineering for classification as the features are automatically learned during the training. DNNs have been applied in many different medical image segmentation tasks. For example, Ref. 11 applied neural networks in segmenting chest radiographs and Ref. 12 used a DNN for predicting fluorescent labels from transmitted-light z stack microscopy images.

Reference 6 conducted a study in which images of fluorescently labeled tissue samples were segmented by extracting image patches around each pixel and feeding them to a deep convolutional neural network for classification. The cell type with the maximum predicted probability was assigned to each pixel so that each pixel was classified to a single class. This kind of technique is known as the sliding window.

Another approach for segmentation is the encoder–decoder network. Original U-Net is one implementation of this kind of network, which takes an image as an input and creates a segmentation for the whole image.^10^ U-Net has been used for many different biological image segmentation tasks. Reference 10 used their DNN (U-Net) in neuronal structures in electron microscopic recording segmentation. Reference 13 demonstrated how a three-dimensional U-Net architecture can be used in delineating radiosensitive organs in head and neck.

The latest DeepLabv3 uses spatial pyramid pooling with different convolutional grid scales to extract rich semantic features. Convolution operations use strides to support large image sizes in situations in which there are scarce memory and computational resources available. It uses a simple decoder module similar to U-Net to capture sharp object boundaries in the segmentation.^14^

A newer approach is the generative adversarial network, which has also been used in segmentation by conditioning the image generation, but they have been seen to produce hallucinated structures that do not exist in the original input images.^15^

It can be difficult to infer information about mutually not exclusive classes.^16^^,^^17^ Segmenting this kind of multilabel images is less common. The class imbalance is also a problem. One way to handle the class imbalance is by oversampling and undersampling the training data. One could also modify the loss function by weighting the classes with varying amounts of data.^11^^,^^16^ The problem has been approached by ensembling many models or a single end-to-end DNN model.^16^

## 3 Methods

### 3.1 Experimental Setup

Cryopreserved PC3 and LNCaP cells were obtained from the University of Turku. The cells were cultured in flasks for 3 days and then were seeded at a density of 5000 cells/well on the top of *in vitro* vascular structures in 96-well plates. Vascular structures were formed as described in Ref. 18 and subsequently decellularized by the method modified from Ref. 19. Cells were maintained in RPMI 1640 supplemented with 1% L-glutamine, 1% penicillin/streptomycin, and 10% fetal bovine serum (Gibco, Thermo Fisher Scientific) in a humidified incubator at 37°C and 5% ${\mathrm{CO}}_{2}$ level. The imaged cells were not exposed to any drugs. The culture period was 14 days in total based on previous experiments.

The images were obtained by acquiring a 2×2 image grid from each well on days 4, 7, 11, and 14 after seeding using noninvasive imaging technology with a Cell-IQ phase-contrast microscope (CM technologies Oy. Tampere, Finland). The time points were chosen to allow the monitoring of the development of the structures seen in the phase-contrast images. In addition, the use of multiple time points allows the different cell types to form their growth patterns, regardless of their different proliferation rates. LNCaP cells are known to proliferate more slowly than PC3 cells. LNCaP cells reached their final growth pattern at the last imaging days. On the contrary, PC3 cells outgrew their wells quickly.

All computations were run on an IBM PowerNV 8335-GTG with two Tesla V100-SXM2 GPUs and 569 GB RAM. The neural networks were created with Python 3.6 and TensorFlow 1.10.^20^

### 3.2 Dataset

The study used 36 full color images (8-bit RGB, resolution 1392×1040) from two prostate-cancer-derived cell lines (PC3 and LNCaP), which were grown on top of vascular structures. PC3 and LNCaP cells were used because they form structures that have defined edges, which can be distinguished by visual inspection.

**Fig. 1:**
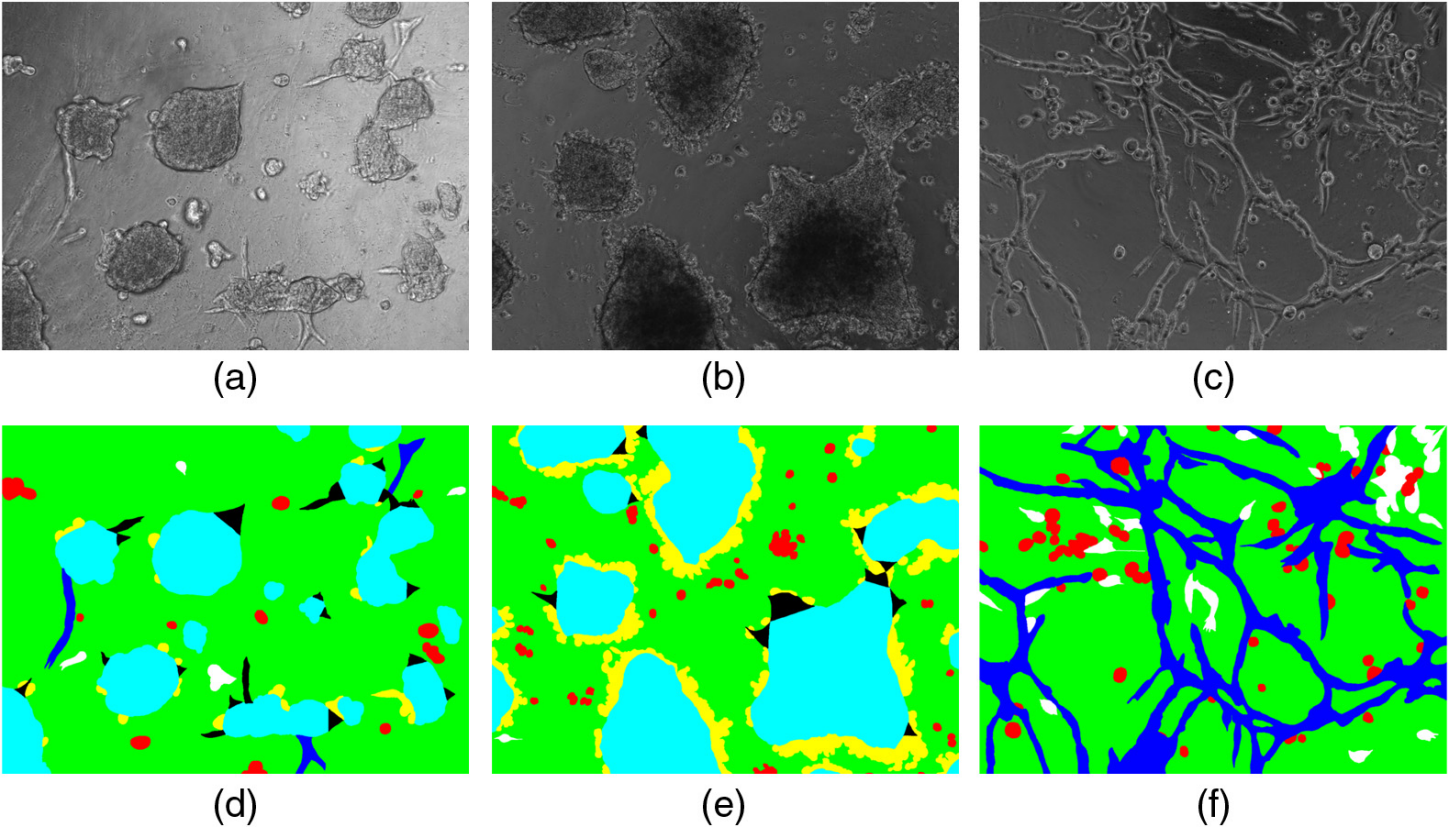
Example training images and their corresponding unprocessed ground-truth images. Multilabel targets are stacked on top of each other. (a) and (b) Images from LNCaP cell line; (c) image from PC3.

The corresponding ground-truth segmentations were created manually by a professional. The ground-truth targets had six classes and the background. The classes could overlap each other; therefore, the images were divided into separate ground-truth images with one target class per image (and the background). Each neural network did binary segmentation to each of these classes. Figure 1 shows three example images from the dataset. All classes are listed below with their corresponding short labels:

• background in green;
• noninvasively growing cells in red (S);
• invasive cells in white (I);
• invasive spikes in black (IP);
• vascular structures in blue (P);
• spheroids LNCaP cells in light blue (O); and
• cell matter around spheroids in yellow (R).

The dataset had some nonuniform markings; thus, correct class information was not available for all images, and some had to be abandoned.

The cells were grown on top of the vascular structures and the invasive and noninvasive cells could overlap each other. This makes the classes mutually not excluding. From the images it could be seen that class P overlapped the most with classes S and I, and I could overlap S. Some images were mostly “empty,” including only a few objects of interest. Some were densely populated with large areas of different structures.

The formation of spheroids, invasive spikes, and the cell matter around them were only observed with LNCaP cells, and distinct vascular networks were mostly observed in PC3 cells. We used images from both cell lines to train neural networks to ensure a sufficient amount of data for training and testing the neural networks.

This limited the number of available images for this study as manual annotation of large images is very time-consuming. Time constraints in the project were the main reason that there are not more images available. The lack of images was compensated for by other techniques in preprocessing, network training, and using multiple networks.

### 3.3 Preprocessing

Single images were too large to be used with U-Net and available GPU memory. Therefore, the images were split into 512×512 subimages. By splitting the images, we obtained a more balanced class distribution.

A total of 34 full color images and their ground truths were used in the training. Only two full color images could be used in testing because the dataset was small and the number of classes was relatively large.

**Fig. 2:**
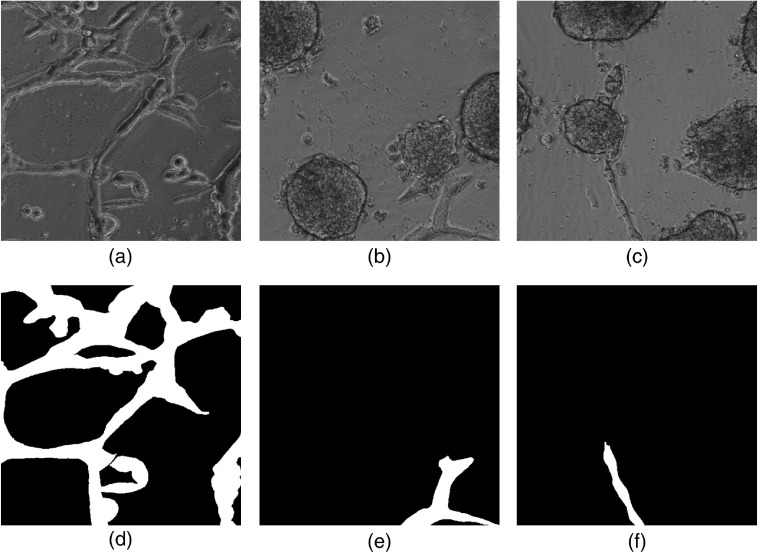
Example training image patches extracted from full images and their corresponding ground-truth images (class P). (a) Image from PC3 cell line; (b) and (c) images from LNCaP.

To create a more balanced training dataset, each image was scanned through and the subimages, including a specific label, were identified. The resulting subimages contained only the target class and background, as depicted in Fig. 2.

Among the group of all subimages for an image with a specific label, at most 20 subimages, including pixels belonging to the class, were selected. These images were the positive samples. The same number of negative samples without the targeted class pixels was also selected. This was repeated for each class. The subimages could overlap each other. The method created many differently aligned views of the same objects of interest. Oversampling or undersampling between classes was not used because each class was predicted by a separately trained neural network. The numbers of subimages for each class are listed in Table 1.

**Table 1:** Numbers of samples used in training.

	P	S	IP	R	O	I
Training samples	1100	660	1239	1020	920	762

### 3.4 Network Architecture

A set of U-Net neural networks was the chosen method. U-Net is a neural network designed for biological image segmentation and for working with small training datasets. Rather than classifying image pixels one by one using a sliding window technique, U-Net inputs and outputs the whole image. This makes the training more efficient. U-Net should be a good choice for the cases in which there are not many training samples available.^10^

U-Net is constructed of convolutional layers and pooling layers that first decrease the resolution of the output. They are followed by upsampling convolutional layers that also concatenate the output in the feature channel axis with the results of the previous layers.

U-Net has a softmax activation function at its last layer and as such is not applicable for classifying mutually not excluding classes. A logistic function could have been used, but the assumption was that one specialized network for a single class would provide more accurate results. Also, the lack of data and the class imbalance encouraged us to train multiple networks, each with specifically selected training images. As a result, each network had two outputs: one for the individual class being predicted and one for everything else.

The created implementation is based on a heavily modified version of U-Net TensorFlow implementation created by Ref. 21.

### 3.5 Training

Based on the earlier tests, cells form two kinds of distinct growth patterns in this *in vitro* method, regardless of the cancer cell line used. Images from both LNCaP and PC3 cell lines were used for training because both cell lines produced structures with similar properties. Also, to increase the number of samples from which to draw subimages for the training, the data from both cell lines were pooled.

To fit the data into the GPU’s memory, the images were first read into the memory of the server and were then fed to the GPU in batches of one or three images (the batch size was one for class P). Additional training examples were generated by augmentation. The data pipeline both rotated and flipped the images horizontally and vertically randomly. The brightness of the images was also randomly changed.

The learning rate, optimizer, and loss function were selected manually based on the literature and experience. Adam^22^ optimizer was used. The weights of the networks were initialized with Glorot and Bengio.^23^ The learning rate was $1\times 10^{-5}$ and set to decay following a staircase function, decreasing every 10,000th global TensorFlow step by the decay rate of 0.9.

The loss function was defined based on Ref. 11 with a regularization term. The purpose of the loss function is to quantify the difference between predictions and ground truths for steering the training of the network. It is defined as

$$C(I,G_{I})=\underset{l\in L}{\sum }r_{l,K}d(I,G_{I})+\lambda \underset{w}{\sum }w^{2},$$ (1)

where the first term calculates the summed loss of the prediction compared with the ground truth, weighted by the class frequency. Here I is the set of images, K is the index of image batch, $G_{I}$ denotes the ground-truth classes of the images, and d is the commonly used softmax cross entropy for the two outputs of the last layer. The outputs are the targeted class and the background. The class is denoted by l belonging to set of classes L, whose size is two for each of the neural networks.

The last term is the L2 regularization over the trainable weights (w). Its purpose is to avoid overfitting the data. Weight factor λ was set to 0.001 because it seemed to reduce the variation of the loss during the training. Pixel-wise batch weighting was utilized by the weighting coefficient $r_{l,K}$:

$$r_{l,K}=\frac{c_{K}}{c_{l,K}},$$ (2)

where $c_{l,K}$ is the number of pixels in the batch K belonging to class l∈L.^11^

The stopping criterion of the training was fixed to 1000 epochs (roughly 5 days of computation time) per neural network. The losses and accuracies of the predictions of the training and test sets were observed with TensorBoard to make sure the models do not overfit during the training. TensorBoard is a tool that can be used to visualize model metrics, such as loss and accuracy during the training. The losses of both training and test data were observed to converge. Because of the lack of data, we did not have a separate validation dataset available. Therefore, the model weights were not selected using the lowest loss for the test data. Optimizing the model to the test data, which is used for calculating the final performance metrics, would introduce bias to the results. The model weights of the 1000th epoch were selected.

### 3.6 Test Image Processing

The full test images had to be split into subimages to be used with the trained networks. For a better view of results, the resulting segmentations had to be postprocessed by combining them back into full-sized images.

Splitting the image in a simple grid was not appropriate because the segmentation created clearly visible artifacts at the edges of subimages. Therefore, a grid of overlapping subimages (30 linearly spaced coordinates in both x and y axes) inside the original image were selected. This resulted in 900 subimages.

All of these subimages were run through the neural network and combined by pixel-wise averaging. The resulting full images show some artifacts, especially at the edges where the pixels are averaged the least.

Other techniques for testing include cross validation and leave-one-out, but they were not used because training the networks for many dataset splits would have been too time-consuming.

### 3.7 Performance Metrics

We used sensitivity (true positive rate or recall), specificity (true negative rate), Dice score (DSC), and area under curve (AUC) metrics. True positives are the predictions that are correctly classified as positive, false positives are wrongly predicted as positives, true negatives are correctly classified as negatives, and false negatives are wrongly predicted as negatives. Manual thresholding was used for calculating these metrics.

Sensitivity corresponds to the proportion of positive data points that are correctly predicted as positive with respect to all positive data points. In other words, higher sensitivity means that fewer positive data points are missed.

Correspondingly, the specificity is the proportion of negative data points that are correctly predicted as negative.

DSC (also known as F1 score) is a similarity index for measuring spatial overlapping of manual ground-truth segmentations and the predictions of automatic methods. DSC ranges from 0 to 1, where 0 indicates no overlap and 1 is a complete overlap.^24^

Receiver operating characteristic (ROC) curve is a method used for assessing the performance of classification algorithms. It is widely used in medical diagnostics. The AUC is the integral of ROC, and one interpretation of it is the probability that the classifier will rank a randomly chosen positive example higher than a randomly chosen negative example. A correct classifier has an ROC above the diagonal and an AUC larger than 0.5.^25^

## 4 Results

Tables 2 and 3 contain the class-wise sensitivity, specificity, and AUC scores for both test images 1 and 2. The results have been calculated from thresholded predictions, where the threshold (0.5) was selected manually based on experience. If we had enough data for a validation dataset, that could have been used with ROCs to select a more optimal operating point. Using the test data to select the thresholds would introduce bias to the results.

**Table 2:** Sensitivity, specificity, AUC with 95% confidence interval, and DSC of image 1. The operating point (threshold) of the ROC was 0:5.

Class	Sens.	Spec.	AUC	DSC
P^a^	0.0	0.998	0.959 [0.959, 0.959]	0.0
S	0.61	0.994	0.978 [0.977, 0.979]	0.533
IP	0.346	0.986	0.867 [0.863, 0.869]	0.261
R	0.893	0.957	0.974 [0.974, 0.975]	0.822
O	0.703	0.983	0.984 [0.983, 0.984]	0.807
I	0.028	0.999	0.785 [0.768, 0.802]	0.03

a Class does not appear in the image.

**Table 3:** Sensitivity, specificity, AUC with 95% confidence interval, and DSC of image 2. The operating point (threshold) of the ROC was 0.5.

Class	Sens.	Spec.	AUC	DSC
P	0.642	0.968	0.949 [0.949, 0.949]	0.741
S	0.591	0.976	0.929 [0.928, 0.930]	0.578
IP^a^	0.0	1.0	0.887 [0.886, 0.887]	0.0
R^a^	0.0	1.0	0.999 [0.999, 0.999]	0.0
O^a^	0.0	1.0	0.999 [0.999, 0.999]	0.0
I	0.275	0.957	0.777 [0.775, 0.779]	0.245

a Class does not appear in the image.

Visualizations of the predictions and their classification errors for test images are illustrated in Sec. 7. Figures 4 and 5 are for image 1. For image 2, the visualizations are shown in Figs. 6 and 7.

### 4.1 Overall Results

**Fig. 3:**
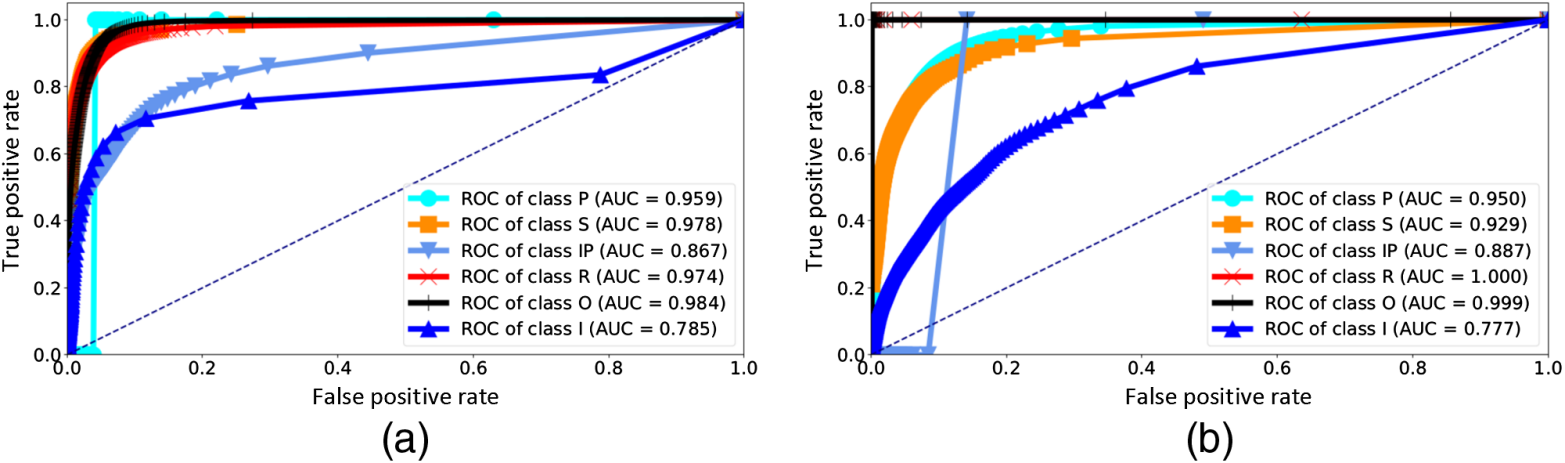
ROCs for the test images (a) 1 and (b) 2.

The AUC scores were relatively high and ranged between 0.777 and 0.984, which implies that the classifiers’ predictions were more often correct than false. The ROCs are shown in Figs. 3(a) and 3(b).

Classes R, O, P, and S all had high sensitivities (0.591 to 0.893). By contrast, classes I and IP had very low sensitivities (0.028 to 0.346). Class I was mainly misclassified as classes P and S.

The specificity was quite high with all of the classes because the models were trained with only two classes: the class in question and the background. Most of the images were filled with the empty background, which led to high specificity.

DSC had high overall variability between classes (0.03 to 0.822). The variability between images could be assessed only with classes S (0.533 to 0.578) and I (0.03 to 0.245). DSC scores were reasonable for P, S, R, and O, as shown in Tables 2 and 3. Classes I and IP had low DSC scores, due to many false negatives.

The models did not make false positive predictions in images that did not include any objects of the targeted class. Therefore, class P had 0.0 sensitivity in image 1. Classes IP, R, and O had 0.0 sensitivity in image 2. This can be seen as very steep ROCs.

### 4.2 Class-Specific Results

#### 4.2.1 Vascular structures

For class P, there was one test image. The predictions did not include many false positives with the specificity of 0.968. Nevertheless, it did not classify the structures correctly where the area was crowded with unclear cells and structures. This affected the sensitivity (0.642).

#### 4.2.2 Invasive cells

Class I’s bad performance shows in the DSC and AUC scores, which were the lowest among all of the classes. Class P was often misclassified as class I, which can be especially seen in Fig. 7(i). The classes share similar properties in their shapes. Class I was not detected in either of the images. It was misclassified as vascular structures (P) and noninvasively growing cells (S), which resulted in many false positives and false negatives.

#### 4.2.3 Invasive spikes

**Fig. 4:**
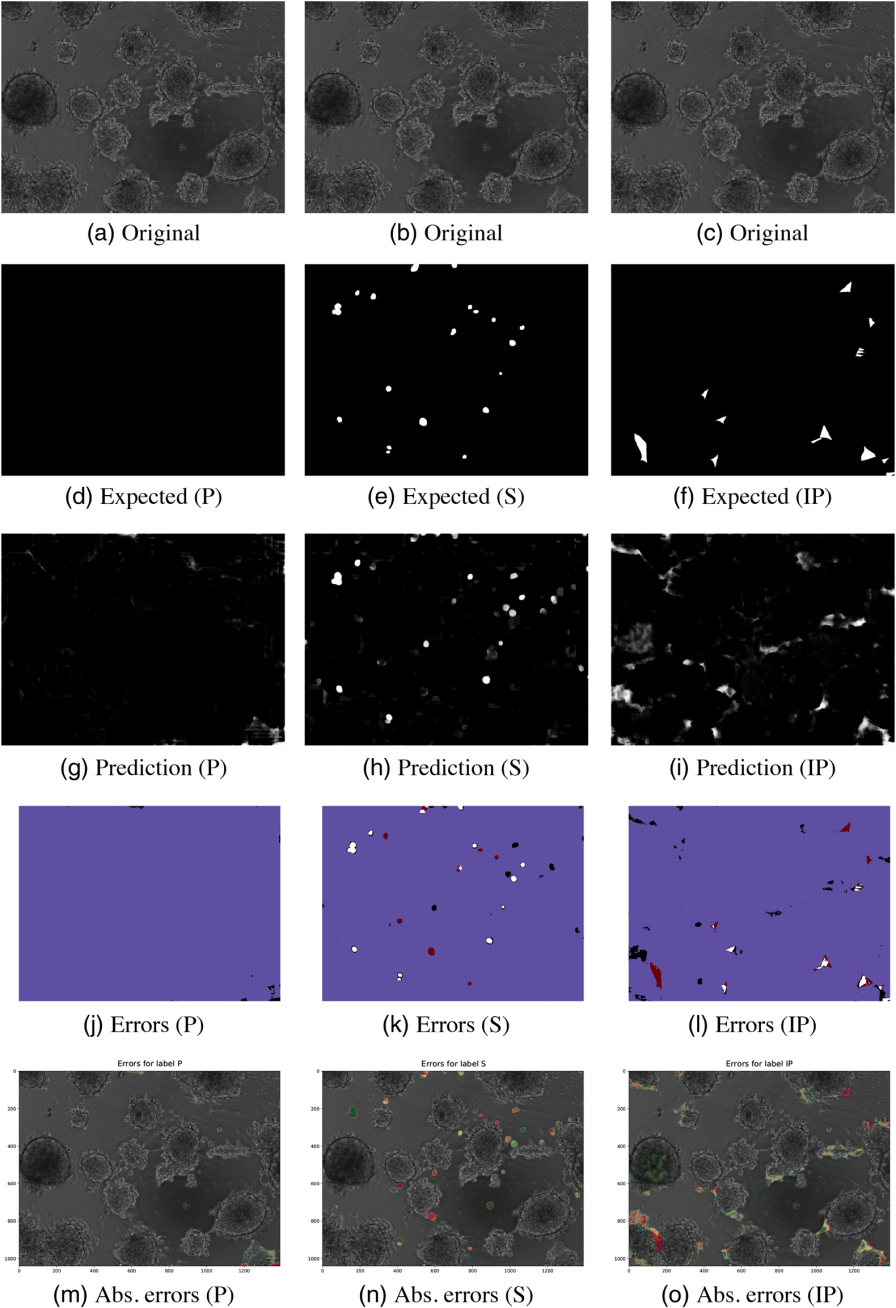
Results for image 1 (PC3) classes P, S, and IP. (a) and (b) The original images, (d)–(f) the ground truths, (g)–(i) the predictions of the models, and (j)–(l) the thresholded pixel classification errors: true positive (white), true negative (blue), false positive (black), and false negative (red). (m)–(o) The absolute errors on the original images (green is more correctly classified and red is wrong).

Invasive spikes had low sensitivity (0.346) and DSC (0.261). The model made some true positive predictions but also many false positive predictions on the surfaces of the spheroids, as seen in Figs. 4(l) and 4(o).

#### 4.2.4 Noninvasive cells

Class S cells had 0.591 sensitivity with image 1 and 0.61 with image 2. In image 1, the problem was distinguishing separate cells from the cell matter around spheroids (class R).

#### 4.2.5 Spheroids

Class O was probably the most recognizable class with distinctive large round shapes. It had good scores with 0.703 for sensitivity and 0.807 for DSC, but suffered from the selected threshold value, which truncated large areas from some of the spheroids.

#### 4.2.6 Cell matter around spheroids

**Fig. 5:**
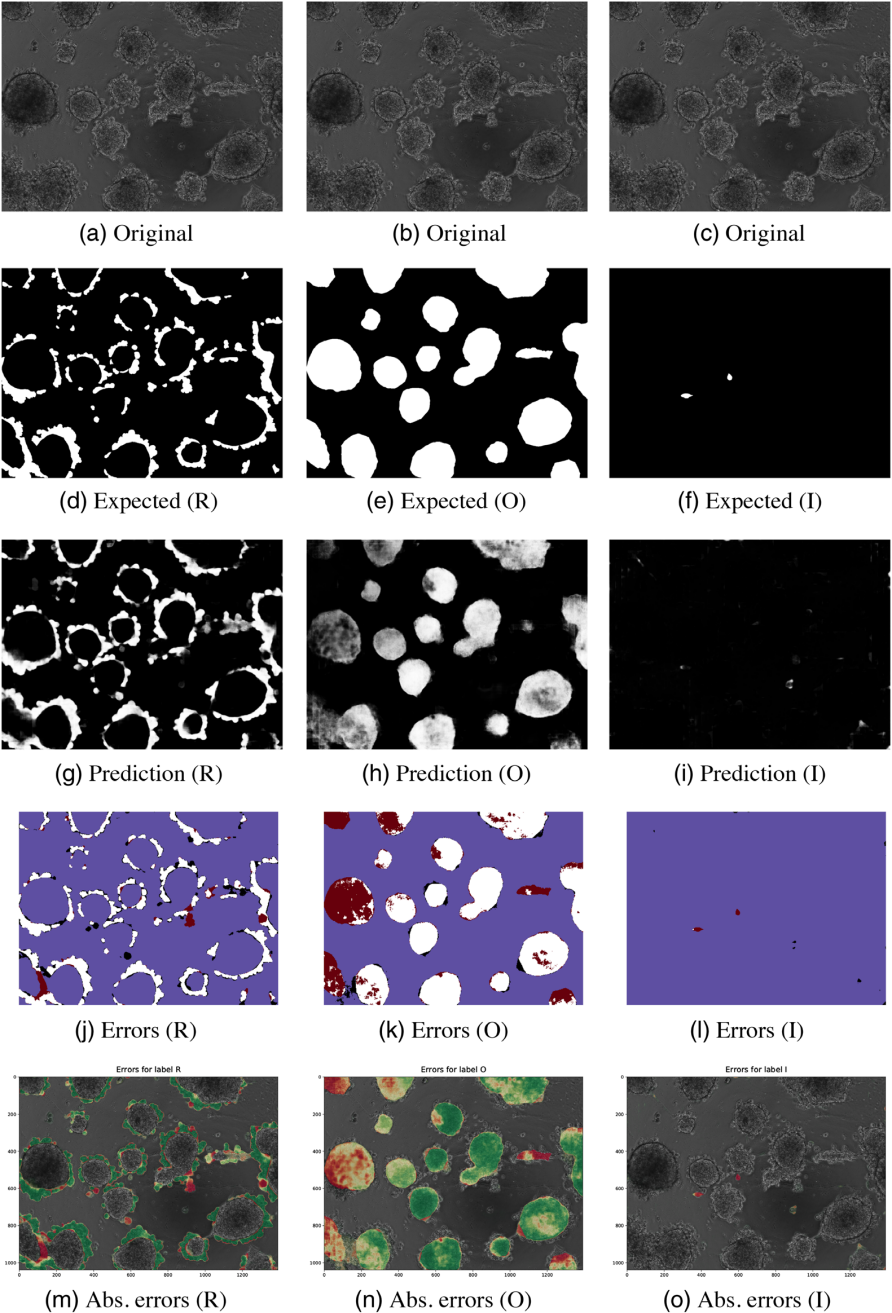
Results for image 1 (PC3) classes R, O, and I. (a) and (b) The original images, (d)–(f) the ground truths, (g)–(i) the predictions of the models, and (j)–(l) the thresholded pixel classification errors: true positive (white), true negative (blue), false positive (black), and false negative (red). (m)–(o) The absolute errors on the original images (green is more correctly classified and red is wrong).

Class R had the highest sensitivity among all classes with 0.893. The AUC (0.974) and DSC (0.822) were both good. Figure 5(j) shows how cell matter is detected mostly correctly, but it had problems with the tight area between spheroids, as it was classified to class O.

## 5 Discussion

Classes R, O, P, and S had very distinctive shapes and clearly defined edges, which led to high sensitivities. Overall, the most evident structures were classified mostly correctly. Classes I and IP had very low sensitivities (0.028 to 0.346), and they were mostly misclassified as other classes.

Some of the invasive spikes (IP) were correctly detected at the edges of spheroids in Fig. 4(l), but the sensitivity (0.346) was low due to mispredicting a few large areas in the ground-truth image [Fig. 4(f)]. The sensitivity was low because the model made many false negative predictions. However, all positive predictions were on the surfaces of the spheroids, which implies that the model has learned that the spikes locate on the edges of the spheroids.

Classes I and IP had very low sensitivity, probably because they shared very similar shapes and textures with other classes. Their training material was not diverse, with a few invasive cells and invasive spikes per image. Many differently aligned subimages were drawn from these examples. Class I was misclassified as P or S and IP as R. They share many similar properties. For example, class I often forms vascular shapes, similar to P, and sharp pointy shapes.

The training material lacked some special cases that occurred in the test images. For example, in image 1 there were two spheroids tightly next to each other with both invasive spikes and cell matter between them. There were no training images for this kind of situation, which could be the reason for the misprediction.

The model trained for class S had quite good scores but struggled with densely populated images. Classes I and R were misclassified as with class S. These classes shared similar properties in size and shape in some cases. In the ground-truth image, some cells around the spheroids were marked as class R and not as noninvasive cells, which in this setting is ambiguous. The cell matter around spheroids is composed of cells. The difference between classes S and R was the distance of the cell from the cluster of cell matter. Taken into account that the markings in the ground-truth images had been made subjectively, the results for S should be taken with a grain of salt in image 1.

Class O would have benefited from more comprehensive data augmentation and optimized threshold selection. As seen in Figs. 5(e) and 5(h), the thresholding has truncated large portions of spheroids where the areas are especially dark. If there would have been enough data for a validation dataset, that data could have been used for selecting a proper threshold using, for example, the high points in the ROCs. More data augmentation with changing image gamma could also have helped in a situation like this.

In terms of the model selection, more exhaustive hyperparameter optimization could have been carried out. The training could have used better weighting for penalizing false positives. Because of the different markings in the data, it was not possible to do class-specific penalization for the background objects. We traded better weighting to a larger number of different training images. However, we could have introduced weighting for all other background classes belonging to a batch. More reliable results would also require comparison with other existing multilabel prediction techniques.

During the experiments, it was noticed that the proliferation rate of the cells greatly influenced how quickly the final growth pattern is reached. Therefore, even though we did not use the primary cells for training the neural network, continuing to image the primary cells for a longer time should be considered, so the final growth pattern would be reached with higher certainty.

The PC3 and LNCaP cells, which were selected for training the neural networks, were not exposed to any drugs, but that will be a goal for future research. Future work would involve using the trained networks to derive data from cancers’ invasion patterns. We could also research if the used cell culture model responds to drugs and can be modified to be a personalized cancer model by utilizing patient-derived primary cells in the future.

## 6 Conclusions

The motivation of this research was to create a personalized medicine model and automate drug efficacy assessment using cancer cell culture microscope images.

Six structures that were thought to play a part in measuring the drug efficiency were identified. Segmenting captured RGB images was the first step to achieving this. The structures were noninvasively growing cells, invasive cells, invasive spikes, vascular structures, spheroids, and cell matter around the spheroids. The structures could overlap each other.

The dataset consisted of 36 RGB images and their ground-truth images for each class. The data were preprocessed and U-Net neural networks were trained to target each of these classes. The method could distinguish vascular structures, cells, spheroids, and cell matter around spheroids in the test images. Some invasive spikes were also detected, but the method could not distinguish the invasive cells.

The limitations of the study were the lack of data and the imbalanced class distribution, which may question the generalizability of the results. The results suggest that more diverse training data were needed. The results are encouraging, taking the amount of data into account, even though confident conclusions cannot be made. Further research is needed.

## Appendix A: Result Images

**Fig. 6:**
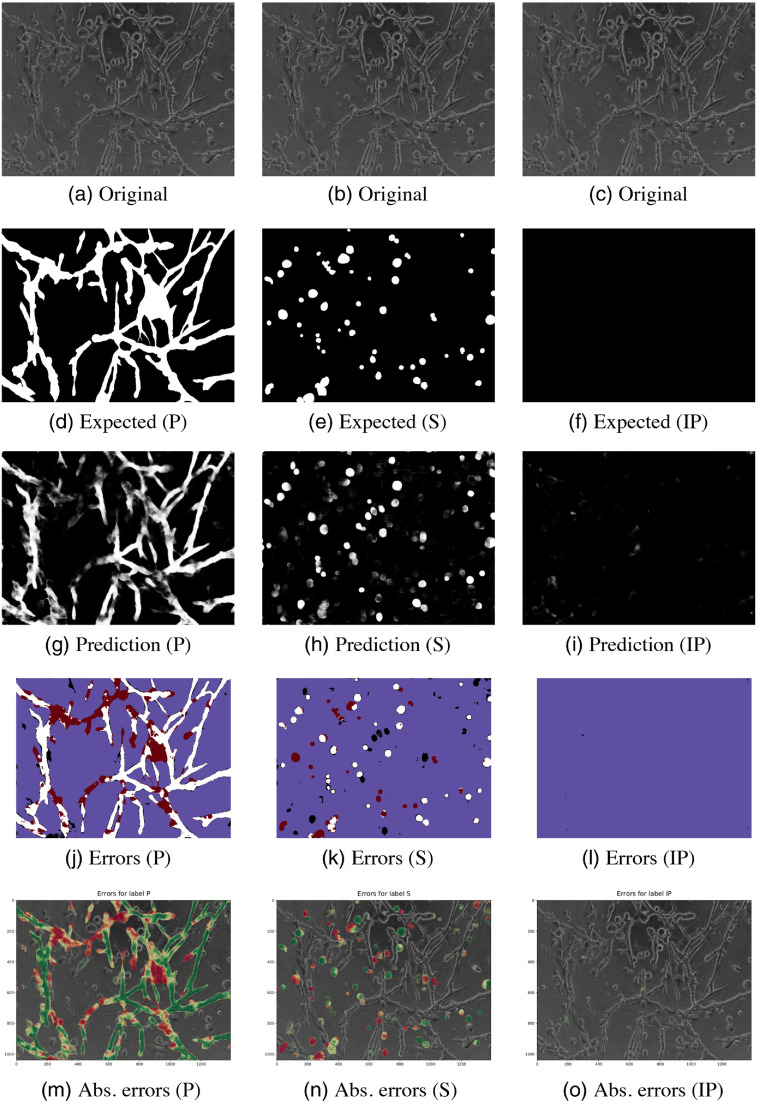
Results for image 2 (LNCaP) classes P, S, and IP. (a) and (b)The original images, (d)–(f) the ground truths, (g)–(i) the predictions of the models, and (j)–(l) the thresholded pixel classification errors: true positive (white), true negative (blue), false positive (black), and false negative (red). (m)–(o) The absolute errors on the original images (green is more correctly classified and red is wrong).

**Fig. 7:**
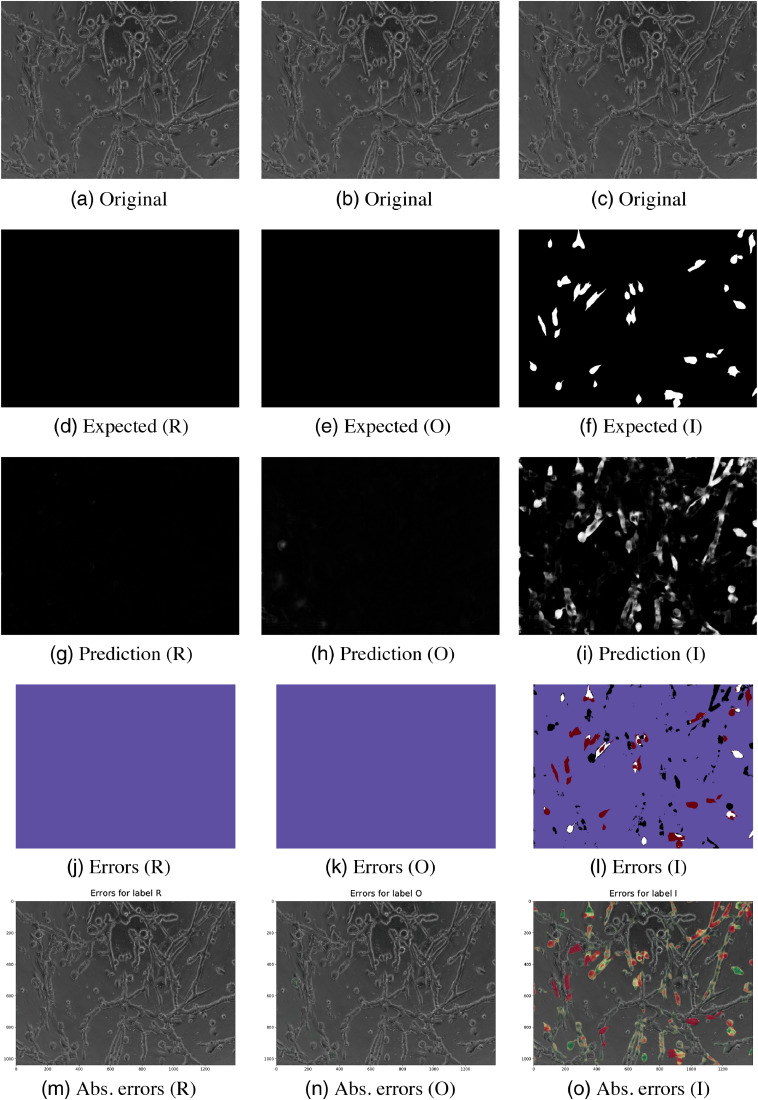
Results for image 2 (LNCaP) classes R, O, and I. (a) and (b) The original images, (d)–(f) the ground truths, (g)–(i) the predictions of the models, and (j)–(l) the thresholded pixel classification errors: true positive (white), true negative (blue), false positive (black), and false negative (red). (m)–(o) The absolute errors on the original images (green is more correctly classified and red is wrong).

The predicted images for two test images are illustrated in Figs. 4–7.
