# Multilabel segmentation of cancer cell culture on vascular structures with deep neural networks (Erratum)

**DOI:** 10.1117/1.JMI.7.2.029801

**Published:** 2020-04-30

**Authors:** Samuli Rahkonen, Emilia Koskinen, Ilkka Pölönen, Tuula Heinonen, Timo Ylikomi, Sami Äyrämö, Matti A. Eskelinen

**Affiliations:** a University of Jyväskylä, Faculty of Information Technology, Jyväskylä, Finland; b Tampere University, Finnish Centre for Alternative Methods, Faculty of Medicine and Health Technology, Tampere, Finland

## Abstract

Corrections to the article, “Multilabel segmentation of cancer cell culture on vascular structures with deep neural networks,” by S. Rahkonen et al.

This article [*J. Med. Imag.* **7**(2), 024001 (2020) doi: 10.1117/1.JMI.7.2.024001] was originally published online on 7 April 2020 with errors in the panel labels for Fig. 6.

The following corrections were made to the originally published version:

1. in Fig 6(h), “Prediction (O)” was corrected to read “Prediction (S)”;
2. in Fig 6(i), “Prediction (I)” was corrected to read “Prediction (IP)”;
3. in Fig 6(j), “Errors (R)” was corrected to read “Errors (P)”;
4. in Fig 6(k), “Errors (O)” was corrected to read “Errors (S)”;
5. in Fig 6(l), “Errors (I)” was corrected to read “Errors (IP)”;
6. in Fig 6(m), “Abs. errors (R)” was corrected to read “Abs. errors (P)”;
7. in Fig 6(n), “Abs. errors (O)” was corrected to read “Abs. errors (S)”; and
8. in Fig 6(o), “Abs. errors (I)” was corrected to read “Abs. errors (IP).”

This article was corrected on 13 April 2020.

